# Association Between PFAS Contamination and Zooplankton Community Structure in the Weihe River, China

**DOI:** 10.3390/toxics14010091

**Published:** 2026-01-19

**Authors:** Jingnan Tan, Haichao Sha, Jinxi Song, Chao Han, Pingping Tian, Le Zhang, Xi Li, Qi Li

**Affiliations:** 1 College of Urban and Environmental Sciences, Northwest University, Xi’an 710127, China; 202332457@stumail.nwu.edu.cn (J.T.); 202310260@stumail.nwu.edu.cn (H.S.); jinxisong@nwu.edu.cn (J.S.); 202410297@stumail.nwu.edu.cn (L.Z.); 2 Xi’an Key Laboratory of Environmental Simulation and Ecological Health in the Yellow River Basin, Xi’an 710127, China; 3 Xi’an Environmental Monitoring Station, Xi’an 710127, China; chaohan_xaepb@163.com (C.H.); 13619212518@163.com (P.T.)

**Keywords:** per- and polyfluoroalkyl substances, zooplankton community, aquatic ecosystem, multivariate coupling effects, PLS-PM

## Abstract

Understanding the structure of zooplankton communities in water contaminated with per- and polyfluoroalkyl substances (PFAS) is essential to the conservation of aquatic biodiversity. This study focused on the Weihe River and systematically characterized the PFAS pollution. By employing environmental DNA metabarcoding, multivariate statistics, and Partial Least Squares Path Modeling (PLS-PM), we systematically analyzed the associations between PFAS and zooplankton within the context of water parameters. The results showed that short-chain PFAS were the dominant PFAS compounds in the Weihe River (accounting for 70.89% of ΣPFAS), and that both PFAS and the zooplankton community exhibited similar spatial patterns. PLS-PM identified a key pathway: water chemistry promoted PFAS accumulation, which in turn exerted taxon-specific effects. Short-chain PFAS were primarily associated with Cercozoa, and path analysis indicated negative relationships, whereas long-chain PFAS were correlated with Ciliophora and Rotifera. Specific taxon within Ciliophora showed potential as bioindicators. Additionally, higher community relative abundance was associated with reduced diversity loss under anthropogenic stress, indicating a potential buffering response. Overall, short-chain PFAS, in combination with water parameters, were associated with higher ecological risk to zooplankton communities. This study highlights the importance of including indirect pathways and taxon-specific responses into risk assessments of emerging contaminants.

## 1. Introduction

Per- and polyfluoroalkyl substances (PFAS) are commonly detected in river environments due to their extensive use and environmental persistence [1]. Despite tightening regulatory restrictions, legacy PFAS, including perfluorooctane sulfonic acid (PFOS) and perfluorooctanoic acid (PFOA), remain widespread in surface waters worldwide [2,3]. A recent global review found that PFAS concentrations in many riverine systems exceed US EPA health advisory levels by factors of 5 to 10 [4], indicating widespread and persistent contamination. The persistent presence of PFAS in freshwater sources, driven by both point sources, such as industrial discharges and wastewater effluents, and non-point sources, such as atmospheric deposition and surface runoff, heightens concerns about their long-term ecological effects. These substances pose potential risks to aquatic organisms through bioaccumulation and trophic transfer, warranting further investigation of their ecological effects on freshwater biological communities.

Aquatic ecosystems are intricate and ever-changing, playing a vital role in ecological processes such as nutrient cycling, energy transfer, and water purification [5]. Recent reviews and meta-analyses reported that concentrations in many rivers exceed the predicted no-effect concentrations (PNECs) for aquatic life [6], raising concerns about the ecological risks posed by PFAS to aquatic ecosystems. Toxicological studies have shown that PFAS can harm aquatic organisms through reproductive toxicity, oxidative stress, and endocrine disruption [7,8], and that sensitive species can experience lethal effects at environmental concentrations [9]. However, previous findings have primarily focused on individual-level endpoints, while community-level impacts remain poorly understood. This knowledge gap may be partly attributed to the fact that PFAS are generally present at much lower environmental concentrations than many conventional pollutants, which have been considered the dominant drivers of aquatic communities. Nevertheless, despite their trace concentrations, PFAS constitute a critical environmental stressor that cannot be overlooked because of their extreme persistence, bioaccumulation potential, and specific toxicity. As research on the ecological effects of PFAS has progressed, attention has shifted toward their community-level consequences. Recent studies have shown that PFAS alter the composition of aquatic communities [10] and that their effects are modulated differently by point sources versus ambient factors [11]. This necessitates a deeper investigation of the responses of specific taxa and the mediating role of environmental context. Zooplankton, as a key trophic link and sensitive bioindicator in aquatic ecosystems [12,13], have shown species-specific toxicity to PFAS [14,15]. However, broader community-level impacts on biodiversity and structure remain unclear. Our study aims to explore these relationships, integrating key water quality parameters to assess the role of the surrounding environment.

The Weihe River is the largest tributary of the Yellow River, and its ecological health is crucial to the entire Yellow River basin. As a representative large freshwater system in northern China, human activities along the Wei River—including industrial, agricultural, and urbanisation processes—exhibit a distinct upstream-to-downstream gradient. The river traverses densely populated urban areas and is subject to significant anthropogenic pressures [16]. Previous studies have reported PFAS contamination risks in this watershed [17]. This research aims to investigate the distribution characteristics of PFAS and their relationship with zooplankton in order to understand ecological effects, thereby providing scientific evidence for protecting this critical aquatic ecosystem.

By capturing information on species composition, environmental DNA (eDNA) offers a novel approach to assess community responses across multiple taxa to PFAS exposure, mediated by water physicochemical factors. Compared to traditional morphological identification methods, eDNA metabarcoding provides higher resolution of how aquatic communities respond to PFAS [18]. Partial least squares path modeling (PLS-PM) is an effective approach for elucidating complex multivariate relationships and quantifying direct and indirect effects [19,20], yet it remains underutilized for investigating the relationship between PFAS and aquatic biota, such as zooplankton. To address this gap and isolate the ecological role of PFAS from broader environmental gradients, this study quantified 14 priority PFAS congeners in the Weihe River. We applied partial least squares path modeling to explicitly control for key background water parameters. This approach allows us to delineate the direct and indirect pathways by which PFAS influence zooplankton communities, providing a more realistic assessment of their in situ ecotoxicological impact. Building on eDNA-derived relative molecular signals to assess ecological responses to environmental stressors, this study focuses on the Weihe River to evaluate the ecological risks posed by PFAS and their associations with zooplankton communities under complex water environmental conditions.

The study investigated environmental stressors, including the occurrence of PFAS, on zooplankton community composition and structure. Specifically, the study aims to (1) characterize the occurrence and spatial distribution of PFAS in October 2023; (2) evaluate relationships among PFAS, environmental variables, and zooplankton community composition and diversity; and (3) construct a PLS-PM to integrate water parameters and reveal key associations between PFAS exposure and zooplankton community dynamics. By investigating and analyzing environmental stressors and biological communities in the field, this research provides preliminary insights into PFAS-related ecological risks.

## 2. Materials and Methods

### 2.1. Study Area and Sample Collection

**Figure 1:**
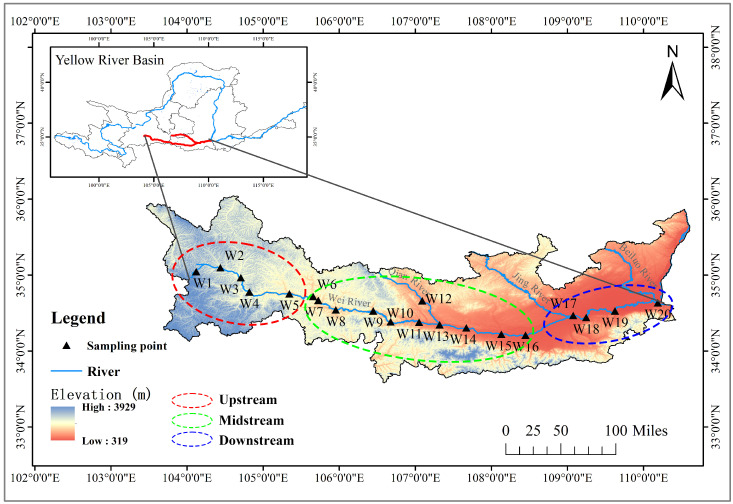
Location of the Weihe River within the Yellow River Basin, showing sampling sites W1–W20 along the mainstem river channel and elevation gradients (319–3929 m a.s.l.).

The main stem of the Weihe River spans 818 km across Gansu and Shaanxi Provinces. In this study, 20 representative sampling sites were selected along the main stem of the Weihe River (Figure 1, Table S1). In October 2023, surface water was collected at a depth of 0.5 m below the water surface using sterile bottles (Thermo Fisher Scientific, Waltham, MA, USA). Depending on the testing requirements, three samples were collected simultaneously at each sampling point: 500 mL of contaminated water for PFAS concentration analysis; 1 L of water for eDNA extraction; and 1 L of water for water parameter determination. Additionally, a 500 mL polypropylene bottle filled with ultrapure water was placed at each sampling point as a field blank to verify sampling quality. A global positioning system (Garmin Legend, Garmin, Olathe, KS, USA) was used during sampling to record geographic coordinates, ensuring accurate and reliable spatial data.

EDNA samples were filtered on the day of collection using 0.45 μm microporous membranes (Merck Millipore, Burlington, MA, USA) and immediately stored at −80 °C for later eDNA extraction. PFAS analysis samples and water quality parameter measurement samples were quickly stored in a 4 °C vehicle-mounted refrigerator after collection and transported to the laboratory once all sampling was completed. The sampling process was strictly adhered to the standard protocol, and all tools were sterilized before use to prevent contamination from external sources.

Water temperature (WT, °C), electrical conductivity (EC, μs/cm), pH, turbidity (NTU), dissolved oxygen (DO, mg/L), total dissolved solids (TDS, mg/L), transparency (SD, m), and oxidation-reduction potential (ORP, mV) were measured on site using a portable water quality analyzer (LH-T600, Lianhua Technology, Beijing, China); total phosphorus (TP, mg/L; HJ/T 103–2003), total nitrogen (TN, mg/L; HJ/T 199–2005), and total organic carbon (TOC, mg/L; HJ 501–2009) were analyzed in the laboratory using national standard methods issued by the China National Institute of Standardization.

### 2.2. PFAS Extraction and Analysis

In this study, 14 PFAS monomers were targeted for analysis, including nine perfluorocarboxylic acids (PFCAs, C4–C12) and five perfluorosulfonic acids (PFSAs, C4–C8) (Table S2). Prior to UPLC-MS/MS analysis, water samples require preparation, including filtration, solid-phase extraction, and concentration. Detailed information on the standards, reagents, and sample preparation procedures used in the experiment can be found in Supporting Information (Text S1, Table S3). Analysis was performed using ultra-performance liquid chromatography coupled with triple-quadrupole tandem mass spectrometry (UPLC-MS/MS TSQ Quantis Plus, Thermo Fisher Scientific, Waltham, MA, USA). Procedural blanks were prepared following the same protocol using 500 mL ultrapure water. To monitor potential laboratory contamination, three procedural blanks and three solvent blanks (chromatographic-grade methanol) were interspersed among all samples. The detailed gradient elution procedure is provided in Text S2, and the instrument parameters are shown in Table S4. An internal standard calibration curve covering a wide concentration range (0.5–100 ng/mL) was constructed. All 14 target compounds exhibited excellent linearity (R^2^ > 0.995) within the tested range (Table S5). The method detection limit (MDL) was determined through the analysis of seven parallel samples at concentrations approximating the estimated detection limit. Detailed information on the MDL and spiked recovery experiments is provided in Table S6. All results were adjusted for procedural and field blanks.

MDL = t_(*n*−1,0.99)_ × SD

where SD represents standard deviation, and t represents the t-value at a 99% confidence level with degrees of freedom (*n* − 1). When the sample size is 7, the t-value is 3.14. Concentrations below the MDL are reported as “<MDL” and calculated as half the MDL in statistical analysis.

### 2.3. DNA Extraction, PCR Sequencing, and Biological Analysis

DNA was extracted using the DNeasy Blood & Tissue Kit (Qiagen GmbH, Hilden, Germany). Blank samples were included as negative controls during extraction to monitor potential contamination. To ensure traceability and minimize systematic error, all samples were processed with the same batch of DNA extraction kits. All extractions were performed in a dedicated eDNA laboratory, where all tools and work surfaces were sterilized prior to use.

Targeted amplification of the 18S rDNA V4 region (18SV4) specific to zooplankton communities was performed on 18 eDNA samples using specific primers for DNA metabarcoding. The primers contained dual-index sequences to enable multiplexed library construction. Protocols for DNA extraction, primer design, and PCR amplification followed the methods described in the referenced literature [9]. A separate sequencing library was generated from the PCR products amplified using each primer set. The library was subsequently sequenced on an Illumina MiSeq platform (Illumina, San Diego, CA, USA) using a MiSeq Reagent Kit v3 (2 × 300 bp for 18SV4).

The USEARCH software (version 11.0.667) developed by Edgar was used to process raw sequencing reads and perform denoising with the UNOISE3 algorithm, separating sequencing errors from true biological variation [21]. High-quality sequences were trimmed to a specific length, and those below the threshold were removed. After quality control, the trimmed sequences were deduplicated to obtain unique sequences, and an operational taxonomic unit (OTU) table was built following denoising. The Seed programme classified zooplankton OTUs by alignment with the GenBank database. OTUs that did not meet the BLAST (version 2.15.0) parameters, had a similarity score <85%, a minimum query coverage <80%, or an E-value > 10^−80^ were excluded to enhance taxonomic annotation accuracy. Annotate OTUs according to the taxonomic standards of the NCBI database. For protozoan OTUs without a defined phylum-level taxonomy, classify them at the class level.

### 2.4. Data Analysis

In this study, “abundance” referred to OTU abundances inferred from eDNA metabarcoding, reflecting relative differences in zooplankton community structure. To assess community structure, Species richness, Shannon’s diversity index, Simpson’s diversity index, and Pielou’s evenness index (α-diversity) were calculated based on OTU abundances, and Bray-Curtis dissimilarity matrices were computed followed by non-metric multidimensional scaling (NMDS) to visualize spatial variations in zooplankton communities, using the “vegan” package in R 4.4.3 [22]. Principal component analysis (PCA) was performed using Origin 2024 to characterize variability in PFAS contamination across river sections. Subsequently, the Kruskal-Wallis test, followed by Dunn’s post hoc test, was conducted in Origin 2024. Linear discriminant analysis effect size (LEfSe) was performed on the Wekemo Bioincloud platform (https://www.bioincloud.tech) to identify significantly discriminative OTUs among different regions [23]. Mantel tests were conducted using the “linkET” package in R 4.4.3 to assess correlations between zooplankton communities and environmental variables. Significant predictors from the Mantel tests were included in a redundancy analysis (RDA) performed in CANOCO version 5.0 [24]. PLS-PM was then developed using the “plspm” package in R 4.4.3 to elucidate multivariate relationships. Environmental variables were log_10_(x + 1) transformed to meet normality assumptions. For all ordination and correlation analyses, individual PFAS compounds detection frequencies below 5% and zooplankton taxa with relative abundances below 2% were excluded.

## 3. Results

### 3.1. Concentrations and Profiles of PFAS

Among the 20 surface water samples, 13 of the 14 target PFAS were detected (Table S2). Except for sodium perfluoroheptane sulfonic acid (PFHpS), which was only detected in sample W17, and sodium perfluoropentane sulfonic acid (PFPeS), which was only in sample W3, the detection rates of the other PFAS ranged from 45% to 100%. The concentrations of ΣPFASs ranged from 4.06 to 23.37 ng/L, with the predominant compounds being perfluorobutanoic acid (PFBA), perfluoropentanoic acid (PFPeA), potassium perfluorobutane sulfonic acid (PFBS), and perfluorohexanoic acid (PFHxA). The average concentration of ΣPFASs in downstream (21.37 ng/L) samples was about four times higher than in upstream samples (4.46 ng/L), showing significant spatial differences in PFAS distribution within the Weihe River.

**Figure 2:**
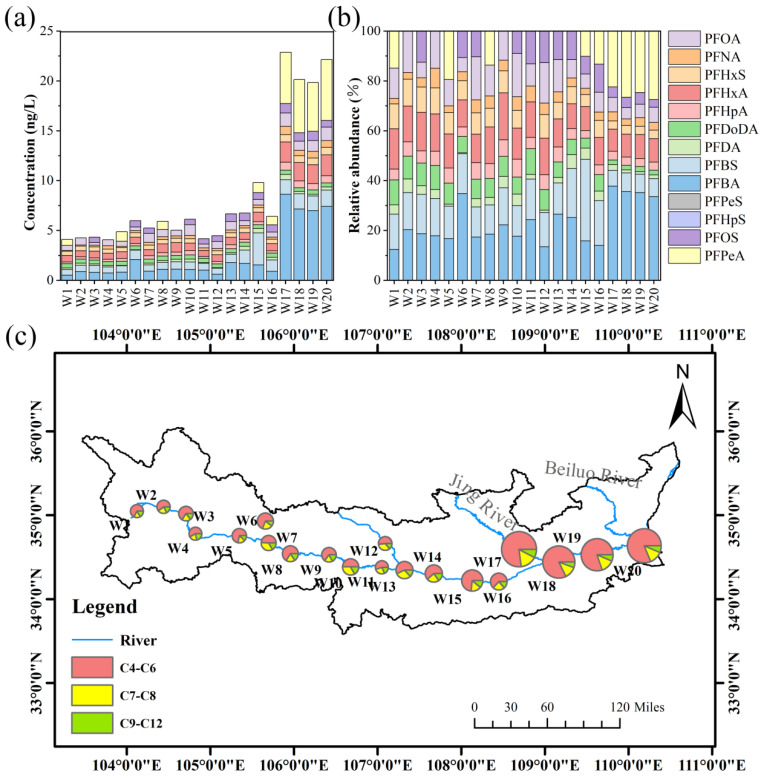
(**a**) Concentration trends of PFAS, (**b**) compositional profiles of PFAS species, and (**c**) spatial distribution of PFAS concentrations by carbon chain length along the Weihe River.

All detected individual PFAS compounds showed a significant increase in distribution downstream (Figure 2). Notably, PFPeA had a detection rate of only 21.42% in the upstream and midstream sections, but was detected in all samples from W17 to W20, with significantly increased concentrations, accounting for 10.17% to 27.53% of the total concentration. PFPeA emerged as the dominant contaminant in the downstream region. Among the 20 sampling points, the predominant compound was C4–C6 PFCAs, accounting for 56.16% of the total concentration, followed by C4–C6 PFSAs (17.73%) and C7–C8 PFCAs (15.23%). Overall, short-chain PFAS made up a larger proportion than long-chain PFAS. The ΣPFCAs concentration (2.66–19.74 ng/L) was higher than the ΣPFSAs concentration (0.99–4.35 ng/L).

### 3.2. Zooplankton Community Structure, Composition, and Diversity

**Figure 3:**
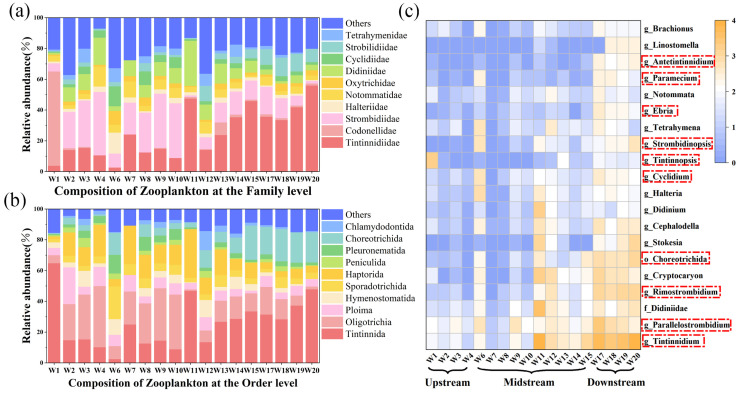
Community composition of zooplankton at the family (**a**) and order (**b**) levels for each site based on OTU abundance (%). The top 20 genera with the highest relative abundance (%) in surface water samples (**c**), in which genera showing significant correlations (*p* < 0.05) with ΣPFASs concentrations are marked with red dashed boxes.

Based on the sequencing results, the composition and relative abundances of zooplankton communities at all sampling points except W5 and W16 were analyzed. After quality filtering and bioinformatics processing of the sequencing library, a total of 117,068 reads associated with zooplankton were obtained, resulting in 185 OTUs. These OTUs represented six taxa, with protozoa being the dominant group, accounting for 155 OTUs (84.24%). The major taxa were Ciliophora (73.37%), followed by Rotifera (13.04%), Choanoflagellata (6.52%), and Cercozoa (4.35%). Notably, the order *Tintinnida* at site W1 was predominantly composed of the family *Codonellidae* (95.15%), which gradually gave way to the dominance of the family *Tintinnidiidae* downstream. The top 20 genera with the highest relative abundances exhibited distinct distribution patterns across the sampling sites, with relative abundances generally higher at downstream sites compared to upstream ones (Figure 3c). However, *Tintinnopsis*, *Brachionus*, and *Halteria* showed a decreasing trend in their relative abundances.

**Figure 4:**
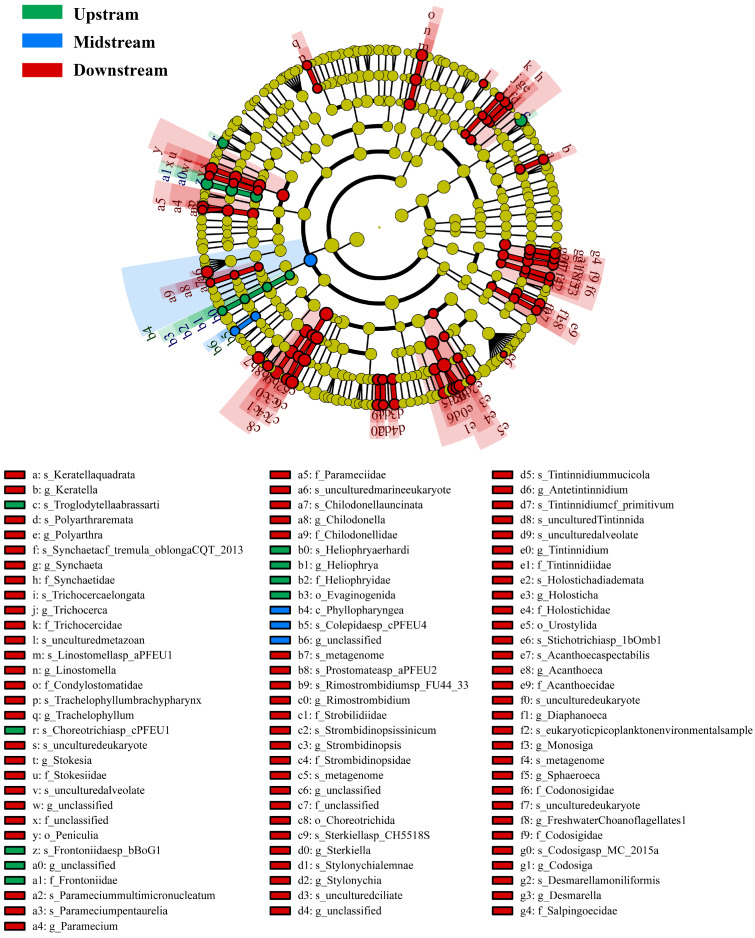
Zooplankton groups with differential abundances in the surface water of the Weihe River. Each ring represents a taxonomic level, from the center to the periphery: Phylum, Class, Order, Family, and Genus. Each circle denotes a taxonomic unit in the dataset, and colored nodes represent taxa with significantly different abundances (green, blue, and yellow indicate the upstream, midstream, and downstream regions, respectively; yellow also denotes no significant difference).

**Figure 5:**
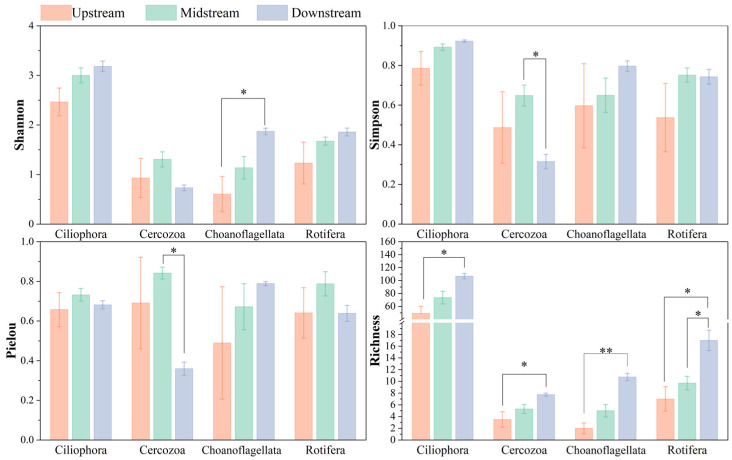
Alpha-diversity of the main taxa (abundance > 2%) of zooplankton in different reaches of the Weihe River (“*” indicates *p* ≤ 0.05; “**” indicates *p* ≤ 0.01).

LEfSe analysis indicated that different sample groups exhibited significantly enriched taxa, with a higher number of dominant taxa found downstream (Figure 4). Ciliophora and Rotifera were the major taxa contributing to these differences. Correlation analysis revealed that the relative abundances of downstream-enriched genera, including *Tintinnidium*, *Stokesia*, *Rimostrombidium*, and *Strombidinopsis*, were significantly positively correlated with PFAS concentrations, particularly short-chain PFAS. Zooplankton community major taxa α-diversity indices across different river segments showed no significant statistical differences; however, they exhibited a clear upstream-to-downstream trend (Figure 5). Species richness across all taxa increased spatially downstream, and communities with higher relative abundance had correspondingly higher species richness. Under normal circumstances, diversity and evenness indices tended to rise with increasing species richness. However, the Cercozoa community at downstream sampling sites was a notable exception: its evenness and Simpson diversity indices decreased significantly. This indicated greater community dominance, reflecting a distinct compositional state compared with midstream communities.

### 3.3. Spatial Variation in PFAS and Zooplankton Community

**Figure 6:**
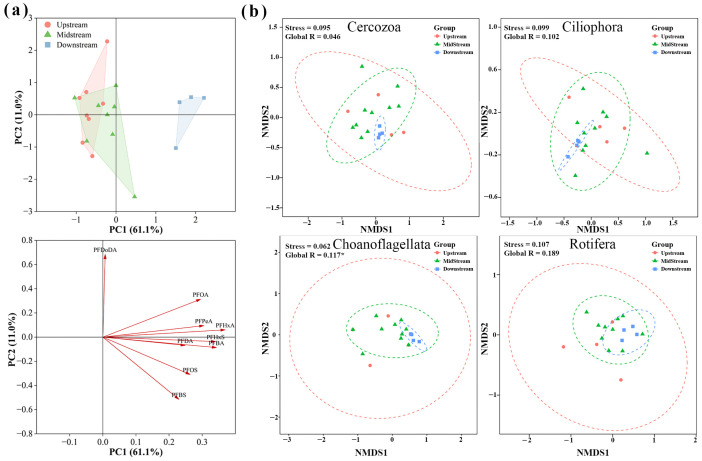
The figure displays the outcomes of PCA of PFAS concentrations (**a**), and NMDS of major zooplankton taxa based on Bray-Curtis distance (**b**) for all stations. (Red, green, and blue dots represent the upstream area (US), midstream area (MS), and downstream area (DS), respectively) (* *p* ≤ 0.05).

Based on topographical features and the distribution of human activities within the Weihe River, the basin was divided into upstream (W1–W5), midstream (W6–W16), and downstream (W17–W20) regions. The PCA plot illustrated the distribution of PFAS samples (Figure 6a). PC1 and PC2 explained 61.1% and 11.0% of the variance, respectively, and the samples showed a clear spatial gradient along the PC1 axis. NMDS ordination of the zooplankton community revealed that the downstream Choanoflagellata assemblage formed a distinct spatial group, with no upstream samples falling within its confidence region (Figure 6b). For Choanoflagellata, permanova confirmed a statistically significant difference in community structure between upstream and downstream reaches (*p* < 0.05). This community spatial pattern coincided with the downstream PFAS concentration gradient.

### 3.4. Correlation Between PFAS, Water Parameters, and Zooplankton Community

Spearman’s rank correlation analyses were conducted to assess relationships among PFAS congeners and physicochemical variables in surface water of the Weihe River, with all variables standardized prior to analysis. Significant positive correlations were observed among several short-chain PFCAs, including PFBA, PFPeA, and PFHxA (r > 0.7, *p* < 0.01), indicating a likely common source or comparable environmental behavior. In contrast, perfluorododecanoic acid (PFDoDA) showed no significant correlation with any other PFAS congener, suggesting a distinct origin or environmental pathway.

Regarding the influence of water parameters, PFAS concentrations were significantly correlated with several key physicochemical parameters. Notably, multiple PFAS compounds showed statistically significant associations with pH, ORP, EC, and TN (Spearman’s r = −0.68 to 0.78, *p* < 0.05), highlighting the role of ambient geochemical conditions in their distribution. Conversely, correlations among the physicochemical parameters were generally weak, except for the significant association between EC and TDS, which was expected.

**Figure 7:**
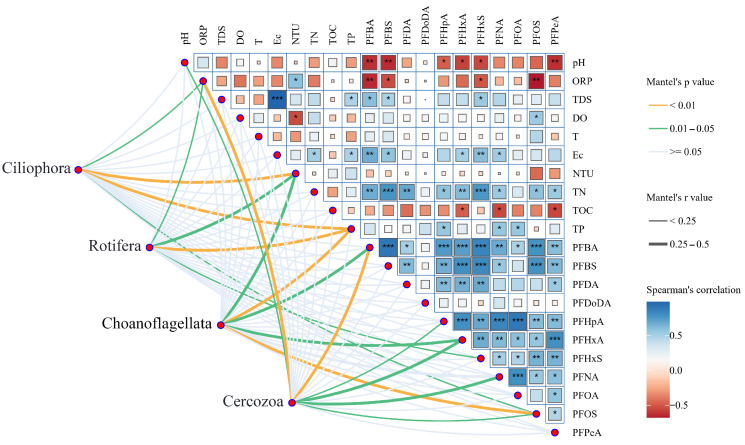
Relationships between zooplankton communities and environmental variables. The network diagram illustrates the results of Mantel tests between different zooplankton taxa (Ciliophora, Rotifera, Choanoflagellata, and Cercozoa) and environmental variables. The width and colour of the connecting lines represent Mantel’s r value and the significance level (*p*-value), respectively. The heatmap shows pairwise correlations among environmental variables. For each pair, the size and colour of a block reflect Spearman’s r value, where blue denotes a positive correlation and red a negative correlation (* *p* ≤ 0.05, ** *p* ≤ 0.01, *** *p* ≤ 0.001).

The results of the Mantel test correlation analysis indicated significant interactions among PFAS, water physicochemical properties, and zooplankton community composition (Figure 7). Significant correlations with the biological community were observed in waters containing PFBA, PFBS, PFHxA, PFOS, ORP, TP, and NTU (*p* < 0.05). Analysis of specific PFAS monomers revealed distinct correlation patterns across zooplankton taxa. The Ciliophora community correlated with long-chain PFAS (PFOS, sodium perfluorohexane sulfonic acid (PFHxS)), whereas the Choanoflagellata and Cercozoa communities correlated with short-chain PFAS (PFBA, PFHxA). Among physicochemical parameters, NTU and TP showed significant correlations with several zooplankton taxa (Mantel’s r = 0.27–0.38, *p* < 0.05). Pairwise Spearman correlation analysis among individual variables further identified significant bivariate associations. The Cercozoa community was jointly correlated with PFBA and ORP, and the Ciliophora community with PFOS and ORP. Additionally, PFBA and PFOS concentrations were each negatively correlated with ORP (Spearman’s r = −0.54, *p* = 0.004). At the taxon level, the strength of association with environmental variables varied. Choanoflagellata and Cercozoa exhibited stronger correlations with PFAS compounds, whereas Ciliophora and Rotifera showed stronger correlations with physicochemical parameters.

Mantel tests indicated that the community composition of Cercozoa, along with its Simpson’s diversity index (Mantel’s r = 0.24–0.40, *p* < 0.05) and species evenness (Mantel’s r = 0.26–0.58, *p* < 0.05), were significantly correlated with profiles of multiple PFAS compounds (Figure S1). Furthermore, within the Choanoflagellata community, the PFOS profile was significantly correlated with several diversity indices, including Shannon’s index, Simpson’s index, and Pielou’s evenness index (Mantel’s r = 0.20–0.37, *p* < 0.05). Regarding water parameters, NTU showed the strongest correlations with zooplankton species richness across communities (Mantel’s r = 0.44–0.46, *p* < 0.05).

**Figure 8:**
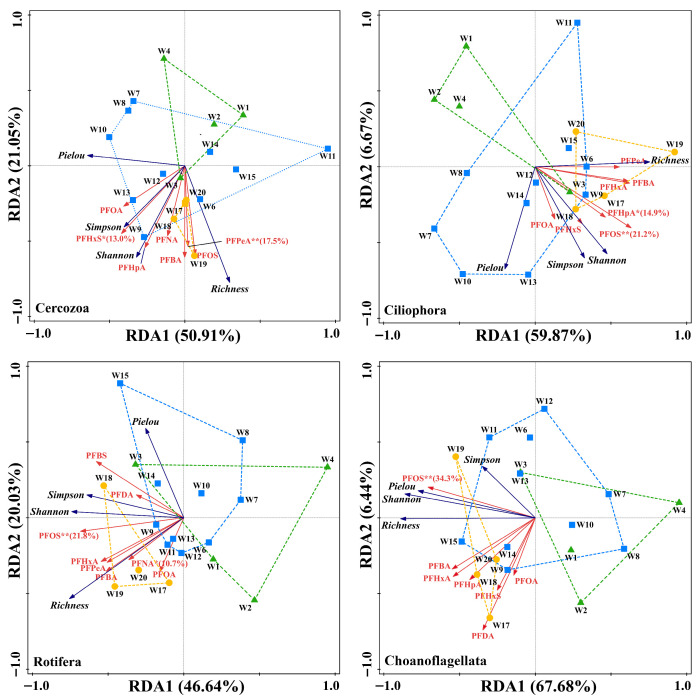
Redundancy Analysis (RDA) of PFAS and zooplankton taxon community diversity, with significant effects, in Weihe River surface water. (Green, blue, and yellow dots represent the upstream area (US), midstream area (MS), and downstream area (DS), respectively.) (“*” indicates *p* ≤ 0.05; “**” indicates *p* ≤ 0.01).

RDA was performed to elucidate the relationship between PFAS and zooplankton community structure (Figure 8). The first two RDA axes explained 66.54% to 74.12% of the total variance, indicating that the selected environmental variables captured a substantial portion of community variation. The ordination biplot showed distinct associations between zooplankton taxa and specific PFAS. The Cercozoa community exhibited the closest correlations with PFPeA (explaining 13.8%) and PFHxS (explaining 17.5%). Conversely, the Ciliophora, Rotifera, and Choanoflagellata communities aligned principally with PFOS (explaining 21.2–34.3%). Vectors representing downstream-enriched short-chain PFAS (PFPeA, PFBA, PFBS) pointed toward higher biodiversity indices but opposed higher evenness indices in the ordination space. At the sample level, downstream sites clustered in regions associated with higher species richness, while midstream sites grouped in areas of higher evenness. Downstream samples were positioned nearer to major PFAS vectors than upstream or midstream samples.

### 3.5. PLS-PM Model Results

**Figure 9:**
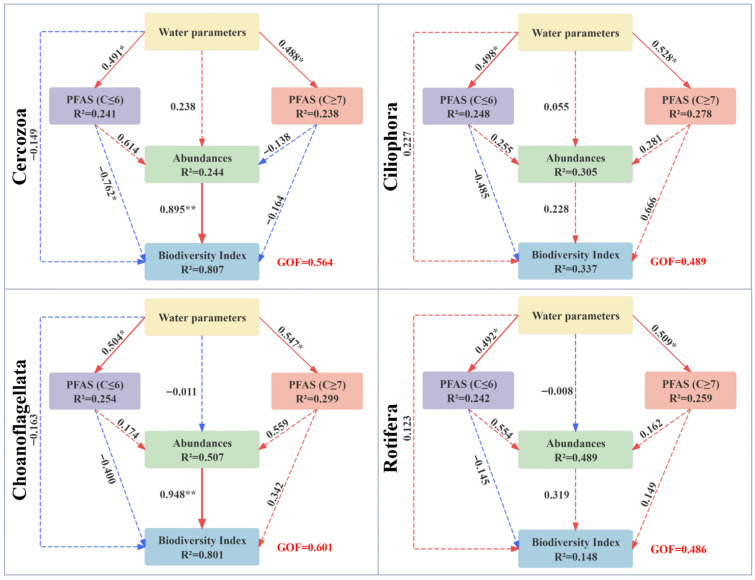
Effects of short- and long-chain PFAS, Water parameters on taxon level zooplankton abundance and biodiversity in the Weihe River are analyzed by Partial Least Squares Path Modeling (PLS-PM). Notes: Blue and red lines represent negative and positive effects, respectively. Solid and dashed lines represent significant and non-significant effects, respectively. (“*” indicates *p* ≤ 0.05; “**” indicates *p* ≤ 0.01).

PLS-PM was used to quantify interactions among relative abundance, biodiversity, water environmental factors, and PFAS contamination (Figure 9). The model showed a satisfactory fit (GoF > 0.45), internal consistency (Cronbach’s α > 0.7), and construct validity (AVE > 0.5). Stability and significance of path coefficients, loadings, and weights were assessed via bootstrapping with 5000 resamples. In the measurement model, EC and ORP were identified as key environmental drivers (loadings 0.836–0.896). The core short-chain PFAS monomers were represented by PFBA (loading 0.980–0.985) and PFHxA (loading 0.987–0.991), while long-chain PFAS were represented by perfluoroheptanoic acid (PFHpA) (loading 0.940–0.973) and perfluoroheptanoic acid (PFNA) (loading 0.939–0.964). The model demonstrated moderate overall explanatory power for variations in PFAS concentrations and community structure. Specifically, it exhibited the highest explanatory power for the biodiversity modules of Choanoflagellata and Cercozoa, with coefficients of determination (R^2^) both exceeding 0.8.

The model estimated a significant positive direct path from the water environment construct to the combined PFAS construct (β = 0.487–0.547, *p* < 0.05). A strong positive direct path from abundance to diversity was also estimated, most notably for Choanoflagellata (β = 0.948, *p* < 0.001) and Cercozoa (β = 0.895, *p* < 0.001). For the Cercozoa community, a significant negative direct path from the Short-chain PFAS construct to diversity was estimated (β = −0.762, *p* < 0.05). In contrast, for the Ciliophora, Rotifera, and Choanoflagellata communities, the Long-chain PFAS construct was associated with positive direct paths to both abundance and diversity, whereas direct paths from the Short-chain PFAS construct were not statistically significant.

Regarding the overall effects, the total standardized effect of the Short-chain PFAS construct on Cercozoa diversity was negative (β = −0.212). This net effect emerged as the strong negative direct path was partially counterbalanced by a positive indirect path via increased abundance (indirect effect β = 0.550). Furthermore, the associations between water environmental factors and zooplankton community metrics were primarily indirect (Figure S2).

## 4. Discussion

### 4.1. PFAS Levels, Composition, and Spatial Distribution

The concentrations of ΣPFASs detected in this study ranged from 4.06 to 23.37 ng/L, with a mean of 8.71 ng/L. These levels were lower than those previously reported in the lower Weihe River tributaries, such as the Beiluo and Qingjian Rivers (ΣPFASs 4.28–372 ng/L, mean 50.1 ng/L) [25], and in the middle reaches of the Yellow River (ΣPFASs 18.4–556.9 ng/L, mean 54.3 ng/L) [26]. Compared with other regions in China, the ΣPFASs concentration in the Weihe River was higher than that in the Yellow River source area on the northwestern Tibetan Plateau (4.25–42.1 pg/L) [27], but lower than that in the Huai River Basin (29.83 ng/L–217.96 ng/L) [28] and the Pearl River Basin (11.8–281 ng/L) [29] in the southeastern region. This spatial variation aligned with the previously reported nationwide gradient of per- and PFAS contamination in China, which correlated with population density and industrial intensity, highlighting anthropogenic pressures as the primary drivers [30,31].

PFAS contamination in surface waters of the Weihe River was dominated by short-chain PFCAs and PFSAs. This finding was consistent with recent results from the Beiluo River, a Weihe River tributary [10]. Compared with earlier findings in the Weihe River [17], the proportion of short-chain PFCAs had increased significantly; meanwhile, the proportion of PFOS declined from 31.80% (mean 6.33 ng/L) to 5.59% (mean 0.49 ng/L), and the proportion of PFOA fell from 11.90% (mean 2.38 ng/L) to 8.29% (mean 0.72 ng/L).

In the present study, significantly elevated concentrations of PFBA and PFPeA were observed in the downstream section of the Weihe River, indicated the influence of localized point-source discharges. The significant correlations among PFAS congeners observed in both PCA and correlation analyses further support the hypothesis of common rather than diffuse sources. This phenomenon may have been attributed to the concentration of intensive industrial activities—such as metal plating, chemical manufacturing, and textile printing and dyeing—in downstream cities, including Xianyang and Weinan [32,33]. In conclusion, the results of this study emphasize that industrial discharges remain one of the primary sources of PFAS pollution in the surface waters of the Weihe River.

This trend mirrored observations in other major Chinese river systems [34,35] and aligned with the global shift toward short-chain alternatives following regulatory restrictions on long-chain PFAS such as PFOA and PFOS [36,37,38,39]. The widespread adoption of these substitutes was evident across diverse regions worldwide [40,41], indicating a broad, industry-driven transition. Spatially, PFAS contamination exhibited a heterogeneous distribution. Specific short-chain compounds, notably PFBA and PFPeA, showed pronounced concentration peaks in the downstream section. This spatial clustering, together with high inter-congener correlations, pointed to inputs from a common source rather than diffuse non-point runoff. Such a pattern is more consistent with industrial point-source emissions [42,43]. Huang et al. suggested that the risk posed by short-chain PFAS in surface water in China might continue to increase [44]. In conclusion, the potential threat to aquatic ecosystems from the continued rise in short-chain PFAS in the Weihe River warranted urgent attention.

### 4.2. Response of Zooplankton Communities to PFAS Pollution

In this study, distinct patterns of change in community composition, abundance, and biodiversity of zooplankton taxa in the Weihe River under the influence of PFAS were observed. Although relative abundances varied among sampling sites, the predominant taxa were similar to those reported in previous studies [45,46], indicating that the basic composition of the zooplankton community remains relatively consistent across the watershed. In contrast to a prior study in the Beiluo River Basin that found no holistic community correlation with PFAS [10], and consistent with more recent reports of negative correlations between PFAS concentrations and β-diversity [11], our analysis identified significant associations between the Choanoflagellata community composition and PFAS levels. This discrepancy likely stemmed from distinct regional environmental contexts. Furthermore, the effects of individual pollutants on overall community metrics might have been limited [47], whereas associations with PFAS were more discernible at specific taxonomic levels and could be masked in broader community or diversity indices. Such taxon-specific sensitivity is likely obscured when analyzing overall community structure or diversity indices. Although differences in zooplankton α-diversity indices among river sections in this study were not entirely significant, except for species richness, subtle variations in community composition remained noteworthy. Consistent with previous findings that Cercozoa were more sensitive to anthropogenic stress than Ciliophora [48,49], we observed pronounced variations in Cercozoa Simpson’s and Pielou’s indices across sites, which were significantly negatively correlated with PFAS concentrations.

In contrast, Ciliophora relative abundance increased along the PFAS gradient from low- to high-concentration sites, while biodiversity showed minimal change. A previous study suggested that these taxa either possess a broader tolerance range or employ a different response strategy compared to other zooplankton [50]. LEfSe analysis identified several Ciliophora genera at downstream sites that were significantly associated with PFAS concentrations. While such taxa have been proposed as bioindicators for general water quality changes [51,52], their associations observed in this study suggested that they may have had relevance as potential indicators in PFAS-contaminated systems. However, the underlying tolerance mechanisms remained unknown, and their value as definitive PFAS indicators required validation through targeted toxicological studies and further field investigation.

### 4.3. Limitations, Unresolved Complexities, and Implications for Risk Assessment

This study provided multi-layered statistical evidence for the association between PFAS contamination and structural changes in the zooplankton community in the surface water of the Weihe River. Among the 14 typical PFAS measured, the short-chain fraction showed a significant negative correlation with the diversity of the Cercozoa community, whereas the long-chain fraction was positively correlated with the diversity of the Ciliophora, Choanoflagellata, and Rotifera communities. These patterns suggested potential taxon-specificity in the interactions between PFAS and different zooplankton taxa. A plausible explanation for this specificity may have involved the coupling of PFAS physicochemical behavior with zooplankton feeding ecology: the hydrophobicity of long-chain PFAS promoted their adsorption to particles, which could have led to predominant exposure for particle-feeding taxa, while the higher aqueous solubility of short-chain PFAS may facilitate more diffuse exposure via the water phase [53,54]. Divergent feeding strategies among taxa could have contributed to differential exposure pathways and community-level responses [55,56]. Therefore, the mechanism by which associations between PFAS and zooplankton communities may have been attributed to the coupling between the structural properties of PFAS and the trophic characteristics of zooplankton taxa.

PLS-PM further delineated these associations from a statistical pathway perspective. The model identified a significant negative direct path between PFAS and diversity, observed exclusively in the Cercozoa community, which had the lowest relative abundance. In contrast, no statistically significant direct paths were detected in other, more abundant and structurally complex taxa. Concurrently, the model estimated a consistent positive direct association between species richness and community biodiversity. Together, these findings suggested that the measurable statistical associations of PFAS may have been modulated by community structural attributes. In simpler, lower relative abundance communities, environmental gradients might have correlated more directly with diversity metrics, whereas in more complex, species-rich communities, potential PFAS effects could have been buffered by internal processes such as species interactions or functional redundancy, thereby being expressed through more indirect pathways or other unmeasured factors [47,57].

It should be emphasized that this study is based on targeted analysis of 14 well-defined PFAS, whereas environmental PFAS exposure encompasses a broader spectrum of precursors, transformation products, and unknown analogues [17,58,59]. Consequently, the reported statistical associations primarily reflected the effects of the measurable PFAS fractions, while the combined pressure from the total PFAS mixture and other related [16,58] unmeasured substances in the environment might have been captured in the models’ unexplained variance. Nevertheless, Mantel test analysis confirmed that the PFAS association with the zooplankton community was independent of conventional water chemistry parameters; despite much higher concentrations of TDS, TN, and TOC, these general parameters showed no significant correlation with community structure. This supported the specificity of the observed ecological signal to the PFAS contamination gradient rather than to non-specific organic or nutrient loads.

In summary, within the established analytical framework, this study demonstrated that the quantifiable PFAS contamination gradient served as an important and specific statistical indicator of zooplankton community changes in the Weihe River. To move from correlative evidence toward understanding the underlying mechanisms and conducting comprehensive risk assessment, future research should implement long-term ecological monitoring to elucidate the spatiotemporal dynamics of PFAS pollution and the lagged and cumulative nature of its ecological effects [46]. Simultaneously, it is essential to integrate targeted PFAS analysis with techniques that capture the total exposure profile, such as the total oxidizable precursor (TOP) assay [60,61] and high-resolution non-target mass spectrometry. Such an integrated approach is essential for accurately apportioning sources, elucidating exposure pathways, and evaluating the collective ecological risk posed by PFAS mixtures in aquatic ecosystems.

## 5. Conclusions

This study assessed PFAS contamination and associated patterns in zooplankton community structure in the Weihe River. Short-chain PFCAs dominated the PFAS profile, and PFBA and PFOS were identified as key stressors driving shifts in zooplankton community structure. Variations in these associations were observed across compounds with different hydrophobicity and among zooplankton taxa with distinct functional traits, with the Cercozoa community showing the strongest negative correlation with short-chain PFAS levels. Notably, specific Ciliophora taxa were enriched at downstream sites and showed significant positive correlations with PFAS concentrations, suggesting their potential utility as bioindicators of contamination. Physicochemical parameters were linked to PFAS distribution patterns and may have indirectly mediated community responses by influencing pollutant bioavailability. Within the community, higher relative abundance was associated with enhanced biodiversity metrics and appeared to attenuate the negative correlations observed between PFAS exposure and diversity. Moreover, by integrating high-throughput sequencing, eDNA, and trait-based approaches, this study established a novel framework for evaluating the ecological effects of emerging contaminants in freshwater systems. Our findings provided critical insights into the ecological effects of PFAS, supporting the development of effective management strategies and the protection of aquatic biodiversity under multi-stressor conditions.

## Data Availability

The original contributions presented in this study are included in the article/Supplementary Material. Further inquiries can be directed to the corresponding authors.

## Supplementary Materials

The following supporting information can be downloaded at: https://www.mdpi.com/article/10.3390/toxics14010091/s1, Text S1: PFAS preparation procedures and reagents; Text S2: Instrument Operating Conditions; Table S1: Information on the sampling sites at Weihe River; Table S2: List of targets perfluorinated compounds monitored, abbreviations, names and chemical formulas; Table S3: PFAS Standard Information; Table S4: mass spectrometry parameters; Table S5: Linear equations and linear correlation coefficients for perfluorinated compounds; Table S6: Method of detection limit (MDL), spiked recoveries, and spiked precision of the individual PFAS; Table S7: Concentrations of 14 PFAS in 20 samples at Weihe River; Table S8: Water parameters in 20 samples at Weihe River; Table S9: Spearman rank correlations among PFAS concentrations in Weihe River; Figure S1: Relationships between biodiversity at the taxa level and environmental variables; Figure S2: Direct and indirect effects of water quality parameters (WP), short- and long-chain PFAS on zooplankton community abundance (a) and diversity (b), as estimated by partial least squares path modeling (PLS-PM).
