# Supplementary material for: Association Between PFAS Contamination and Zooplankton Community Structure in the Weihe River, China
Source: Toxics. 2026 Jan 19;14(1):91. doi: 10.3390/toxics14010091 (PMC12846284; doi:10.3390/toxics14010091)
Supplement: Supplementary file 1 [file toxics-14-00091-s001.zip › toxics-4065689-supplementary.pdf]

Supporting Information

Association between PFAS Contamination and Zooplankton Community Structure in the Weihe River, China

Jingnan Tan [a,b,1], Haichao Sha [a,b], Jinxi Song [a,b], Chao Han [c], Pingping Tian [c], Le Zhang [a,b], Xi Li [a,b,*] and Qi Li [a,b*]

[a] *College of Urban and Environmental Sciences, Northwest University, Xi'an, 710127，China*

[b] *Xi'an Key Laboratory of Environmental Simulation and Ecological Health in the Yellow River Basin, Xi'an, 710127，China*

[c] *Xi'an Environmental Monitoring Station, Xi'an, 710127，China*

Correspondence:

Prof. Qi Li, Tel.: +86 29 88308427; fax: +86 29 88308427. E-mail address: qili726@nwu.edu.cn (Q. Li);

Dr. Xi Li, Tel.: +86 29 88308427; fax: +86 29 88308427. E-mail address: xili@nwu.edu.cn (X. Li).

The supporting information includes 17 pages (2 Text, 9 Tables, and 2 Figures).

Support Information Captions:

Text S1. PFAS preparation procedures and reagents.

Text S2. Instrument Operating Conditions.

Table S1. Information on the sampling sites at Weihe River.

Table S2. List of targets perfluorinated compounds monitored, abbreviations, names and chemical formulas.

Table S3. PFAS Standard Information.

Table S4. Mass spectrometry parameters.

Table S5. Linear equations and linear correlation coefficients for perfluorinated compounds.

Table S6. Method of detection limit (MDL), spiked recoveries, and spiked precision of the individual PFAS.

Table S7. Concentrations of 14 PFAS in 20 samples at Weihe River.

Table S8. Water parameters in 20 samples at Weihe River.

Table S9. Spearman rank correlations among PFAS concentrations in Weihe River.

Fig. S1. Relationships between biodiversity at the taxa level and environmental variables.

Fig. S2. Direct and indirect effects of water quality parameters (WP), short- and long-chain PFAS on zooplankton community abundance (a) and diversity(b), as estimated by partial least squares path modeling (PLS-PM).

Supplementary text

Text S1. PFAS preparation procedures and reagents.

1) Standards and reagents

Methanol was chromatographic grade, and acetic acid, ammonium acetate, and ammonia solution were of analytical grade, all obtained from Merck Millipore (Massachusetts, USA). Oasis WAX solid-phase extraction cartridges (6 cc/150 mg) were purchased from Waters (Massachusetts, USA). Ultrapure water was produced using a Milli-Q Advantage A10 system (Merck Millipore, Massachusetts, USA). The solid-phase extraction device (LC-SPE-24+) and nitrogen blowdown instrument (LC-DCY-12GP) were both purchased from Lichen Scientific Instruments Co., Ltd. (Zhejiang, China).

The isotope internal standard and standard reserve solutions were procured from AccuStandard (see Table S3 for details). Prior to the experiment, an appropriate volume of the internal standard reserve solution was transferred and diluted with methanol to a concentration of 0.200 μg/mL for subsequent use.

Analysis was performed using ultra-performance liquid chromatography coupled with triple-quadrupole tandem mass spectrometry (UPLC-MS/MS TSQ Quantis Plus, Thermo Fisher Scientific, Massachusetts, USA). Accucore™ C18 HPLC columns were purchased from Thermo Fisher Scientific (No. 17126-052130, 50 mm×2.1mm, Thermo Fisher Scientific, Massachusetts, USA).

2) PFAS preparation procedures

50 μL of the extraction internal standard solution was added to 500 mL of the water sample, and the mixture was thoroughly agitated. The sample was then filtered through a water filtration apparatus (FS300F-T5, Sciencetool, Shanghai, China) using a 0.45 μm glass fiber filter. The SPE column was activated sequentially with 5 mL of

0.1% ammonia-methanol solution, followed by 7 mL of methanol and 10 mL of ultrapure water. The sample was loaded at a flow rate of 3 mL/min. After loading, the cartridge was rinsed with 5 mL of ammonium acetate solution (25 mmol/L), followed by 12 mL of ultrapure water. Residual liquid on the cartridge wall was removed using a vacuum pump, and the cartridge was then dried for 15 minutes. The target compounds enriched on the SPE cartridge were eluted sequentially with 5 mL of methanol, followed by 7 mL of 0.1% ammonia solution in methanol. The eluate was collected in 15-mL polypropylene (PP) centrifuge tubes and concentrated to 0.1 mL under a gentle stream of nitrogen gas. The concentrate was diluted to 1 mL with methanol, vortex-mixed thoroughly, and filtered through a 0.22 μm PP needle filter (NEWSTAR, Hangzhou, China) into an autosampler vial. Finally, 50 μL of the internal standard was added and mixed thoroughly.

Text S2. Instrument Operating Conditions.

The column temperature was maintained at 40°C with an injection volume of 2 μL. Mobile phase A consisted of 5 mmol/L ammonium acetate aqueous solution, and mobile phase B was methanol, delivered at a flow rate of 300 μL/min. The gradient program initiated with 80% A, maintained for 1.5 min, decreased to 10% A over 7.5 min (1.5–9 min), held for 4 min (9–13 min), and returned to initial conditions (80% A) from 13.1 to 15 min. Mass spectrometric analysis was performed using electrospray ionization (ESI) in negative ion mode with multiple reaction monitoring (MRM). Negative ion voltage at 1500 V, sheath gas at 45 Arb, auxiliary gas at 10 Arb, ion transfer tube temperature maintained at 300°C, and evaporator temperature set to 450°C. Detailed mass spectrometry parameters are summarized in **Table S4.**

Supplementary tables

Table S1. Information on the sampling sites at Weihe River.

Site	Longitudes(°E)	Latitude(°N)	Elevation(m)
W1	104.120043	35.047672	2215.500
W2	104.438987	35.099183	1837.310
W3	104.706937	34.967473	1620.510
W4	104.818819	34.778561	1476.800
W5	105.345457	34.755152	1224.670
W6	105.656472	34.719661	1127.440
W7	105.725052	34.669427	1095.130
W8	105.955341	34.542650	1032.100
W9	106.447127	34.529232	875.200
W10	106.675993	34.384964	745.150
W11	107.050113	34.379879	570.520
W12	107.090522	34.663155	692.220
W13	107.319585	34.346770	514.810
W14	107.670552	34.306226	463.120
W15	108.131042	34.221676	385.410
W16	108.448686	34.209741	365.540
W17	109.074785	34.469255	327.580
W18	109.246725	34.442990	346.352
W19	109.624343	34.528843	309.740
W20	110.188421	34.636412	329.654

Table S2. List of targets perfluorinated compounds monitored, abbreviations, names and chemical formulas.

Abbreviation	CAS number	Name	Chemical formula
PFBA	375-22-4	perfluorobutanoic acid	$C_4HF_7O_2$
PFPeA	2706-90-3	perfluoropentanoic acid	$C_5HF_9O_2$
PFHxA	307-24-4	perfluorohexanoic acid	$C_6HF_{11}O_2$
PFHpA	375-85-9	perfluoroheptanoic acid	$C_7HF_{13}O_2$
PFOA	335-67-1	perfluorooctanoic acid	$C_8HF_{15}O_2$
PFNA	375-95-1	perfluorononanoic acid	$C_9HF_{17}O_2$
PFDA	335-76-2	perfluorodecanoic acid	$C_{10}HF_{19}O_2$
PFBS	29420-49-3	potassium perfluorobutane sulfonic acid	$C_4F_9KO_3S$
PFPeS	630402-22-1	sodium perfluoropentane sulfonic acid	$C_5F_{11}SO_3Na$
PFHxS	82382-12-5	sodium perfluorohexane sulfonic acid	$C_6F_{13}NaO_3S$
PFHpS	375-92-8	sodium perfluoroheptane sulfonic acid	$C_7H_{15}FNaO_3S$
PFOS	4021-47-0	sodium perfluorooctane sulfonic acid	$C_8F_{17}NaO_3S$
PFUnDA	2058-94-8	Perfluoroundecanoic acid	$C_{11}HF_{21}O_2$
PFDoDA	307-55-1	Perfluorododecanoic acid	$C_{12}HF_{23}O_2$

Table S3. PFAS Standard Information.

Name	Specifications	Manufacturer
PFBA	100µg/mL	AccuStandard
PFPeA	100µg/mL	AccuStandard
PFBS	50.0 µg/mL	AccuStandard
PFHxA	100µg/mL	AccuStandard
PFPeS	100µg/mL	AccuStandard
PFHpA	100µg/mL	AccuStandard
PFHxS	100µg/mL	AccuStandard
PFOA	100µg/mL	AccuStandard
PFHpS	100µg/mL	AccuStandard
PFNA	100µg/mL	AccuStandard
PFOS	100µg/mL	AccuStandard
PFDA	100µg/mL	AccuStandard
PFUnDA	100µg/mL	AccuStandard
PFDoDA	100µg/mL	AccuStandard
MPFBA	50.0 µg/mL	AccuStandard
MPFOS	50.0 µg/mL	AccuStandard
MPFDA	50.0 µg/mL	AccuStandard
MPFOA	50.0 µg/mL	AccuStandard

Table S4. Mass spectrometry parameters.

ID	Compound	Retention period (min)	Parent ion (m/z)	Daughter ion (m/z)	Crash voltage (V)	Lens Voltage (V)	Source debris voltage (V)
1	MPFBA	3.20	216.99	172.00	9.00	69	0
2	MPFOS	8.37	502.85	79.92	40.44	128	0
3	MPFDA	8.85	514.88	218.88	17.53	95	0
			514.88	469.97	10.01	95	0
4	MPFOA	7.74	416.88	171.88	18.25	258	49
			416.88	371.92	16.10	258	49
5	PFBA	3.20	212.90	168.99	10.00	55	30
6	PFPeA	4.81	262.87	218.89	10.00	90	33
7	PFBS	5.10	298.88	79.80	34.86	142	0
			298.88	98.88	30.56	142	0
			298.88	168.88	22.83	142	0
8	PFHxA	6.06	312.87	118.92	20.00	101	34.7
			312.87	268.99	10.23	101	34.7
9	PFPeS	6.21	349.10	79.92	36.00	169	0
			349.10	98.92	32.00	169	0
10	PFHpA	7.00	362.90	168.99	15.70	118	35
			362.90	318.92	10.23	118	35
11	PFHxS	7.08	398.89	79.95	37.37	128	0
			398.89	98.92	32.71	128	0
1	PFOA	7.74	412.87	168.99	16.14	128	35
			412.87	368.92	10.20	128	35
13	PFHpS	7.78	449.00	79.90	44.00	213	0
			449.00	169.00	32.00	213	0
14	PFNA	8.37	462.87	218.92	15.10	128	35
			462.87	418.99	10.23	128	35
15	PFOS	8.37	498.85	79.92	51.00	128	0
			498.85	98.92	45.00	128	0
16	PFDA	8.87	512.87	268.92	17.00	137	37
			512.87	468.99	10.23	137	37
17	PFUnDA	9.29	562.85	318.96	16.00	128	34.7
			562.85	518.92	10.23	128	34.7
18	PFDoDA	9.65	612.97	268.88	19.32	112	0
			612.97	568.88	11.02	112	0

Table S5. Linear equations and linear correlation coefficients for perfluorinated compounds.

**Compound**	**linear equation**	**correlation coefficient (R^2)**
PFBA	Y=1.126e0x+8.12e-1	0.997
PFPeA	Y=5.072e-1x+1.134e-1	0.995
PFBS	Y=5.008e0x+1.055e0	0.999
PFHxA	Y=7.679e01x+2.045e-1	0.997
PFPeS	Y=4.66e0x+2.894e0	0.998
PFHpA	Y=1.187e0x-1.948e-1	0.996
PFHxS	Y=4.19e0x+3.092e0	0.999
PFOA	Y=1.196e0x+4.37e-1	0.996
PFHpS	Y=3.923e0x+2.48e0	0.998
PFNA	Y=1.532e0x+2.62e0	0.995
PFOS	Y=2.547e0x+2.094e0	0.996
PFDA	Y=2.463e0x+1.874e0	0.998
PFUnDA	Y=4.834e0x+4.172e0	0.996
PFDoDA	Y=5.198e1x+3.708e1	0.996

Table S6. Method of detection limit (MDL), spiked recoveries, and spiked precision of the individual PFAS.

Compounds	MDL (ng/L)	Recovery (%, Mean±RSD, n=6)	Method precision (ng/L, Mean±RSD, n=6)
PFBA	0.7	1.06±0.102	42.33±9.7
PFPeA	0.4	1.08±0.086	43.26±8
PFBS	0.7	1.01±0.123	40.52±12.1
PFHxA	0.5	1.05±0.095	42.13±9
PFPeS	0.6	1.05±0.167	42.12±15.8
PFHpA	0.4	0.972±0.138	38.88±14.2
PFHxS	0.7	1.06±0.065	42.38±6.1
PFOA	0.4	1.06±0.139	42.57±13
PFHpS	0.6	1.1±0.077	44.17±7
PFOS	0.6	0.976±0.145	39.06±14.8
PFNA	0.7	0.823±0.135	32.91±16.4
PFDA	0.4	0.692±0.066	27.69±9.5
PFUnDA	0.3	0.643±0.045	25.71±7
PFDoDA	0.5	0.668±0.045	26.73±6.8

Table S7. Concentrations of 14 PFAS in 20 samples at Weihe River.

Sample (ng L^{-1})	PFBA	PFBS	PFDA	PFDoDA	PFHpA	PFHpS	PFHxA	PFHxS	PFNA	PFOA	PFOS	PFPeA	PFPeS	PFUnDA
W1	0.51	0.58	0.15	0.41	0.18	0.00	0.66	0.41	0.09	0.50	0.00	0.61	0.00	0.00
W2	0.86	0.63	0.23	0.39	0.25	0.00	0.61	0.46	0.12	0.71	0.00	0.00	0.00	0.00
W3	0.81	0.68	0.15	0.39	0.21	0.00	0.67	0.44	0.16	0.27	0.54	0.00	0.70	0.00
W4	0.72	0.61	0.15	0.39	0.23	0.00	0.61	0.42	0.32	0.61	0.00	0.00	0.00	0.00
W5	0.82	0.63	0.04	0.41	0.29	0.00	0.66	0.42	0.13	0.52	0.00	0.95	0.00	0.00
W6	2.07	0.96	0.03	0.39	0.23	0.00	0.65	0.46	0.21	0.34	0.63	0.00	0.00	0.00
W7	0.91	0.63	0.18	0.40	0.35	0.00	0.60	0.45	0.27	0.90	0.54	0.00	0.00	0.00
W8	1.10	0.69	0.17	0.46	0.35	0.00	0.87	0.44	0.29	0.73	0.00	0.81	0.00	0.00
W9	1.12	0.75	0.23	0.40	0.33	0.00	0.95	0.44	0.22	0.59	0.00	0.00	0.00	0.00
W10	1.08	0.75	0.28	0.42	0.44	0.00	0.77	0.43	0.34	1.06	0.55	0.00	0.00	0.00
W11	1.02	0.68	0.08	0.43	0.19	0.00	0.60	0.00	0.27	0.37	0.55	0.00	0.00	0.00
W12	0.60	0.62	0.04	0.37	0.26	0.00	0.66	0.42	0.21	0.72	0.57	0.00	0.00	0.00
W13	1.77	0.83	0.15	0.41	0.40	0.00	0.75	0.44	0.28	0.86	0.76	0.00	0.00	0.00
W14	1.70	1.33	0.37	0.42	0.29	0.00	0.67	0.46	0.23	0.50	0.78	0.00	0.00	0.00
W15	1.55	3.20	0.44	0.39	0.30	0.00	0.95	0.46	0.25	0.56	0.69	1.00	0.00	0.00
W16	0.89	1.15	0.25	0.42	0.25	0.00	0.70	0.44	0.21	0.51	0.72	0.85	0.00	0.00
W17	8.64	1.44	0.54	0.41	0.80	0.51	2.04	0.74	0.84	1.33	0.95	5.13	0.00	0.00
W18	7.15	1.52	0.24	0.37	0.67	0.00	1.85	0.70	0.53	0.92	0.84	5.35	0.00	0.00
W19	6.98	1.46	0.20	0.42	0.64	0.00	1.90	0.66	0.66	1.08	0.94	4.89	0.00	0.00
W20	7.42	1.61	0.32	0.41	0.73	0.00	2.09	0.75	0.67	1.35	0.68	6.09	0.00	0.00

Table S8. Water parameters in 20 samples at Weihe River.

Sample	pH	ORP (mV)	TDS (mg/L)	DO (mg/L)	WT (℃)	Ec (μs/cm)	SD (m)	NTU	TN (mg/L)	TOC (mg/L)	TP (mg/L)
W1	8.32	177.90	144.00	8.81	10.30	224.00	0.315	21.30	0.57	9.44	0.03
W2	8.43	195.50	287.00	8.49	13.10	454.00	0.103	194.00	2.84	14.16	0.03
W3	8.46	190.40	495.00	9.48	10.00	723.00	0.065	OVER RANGE	4.40	12.28	0.03
W4	8.49	201.20	309.00	8.98	14.10	500.00	0.078	324.00	3.87	9.90	0.03
W5	8.39	191.70	333.00	9.66	11.30	502.00	0.063	544.00	5.11	13.75	0.04
W6	8.26	101.10	955.00	8.13	15.50	1565.00	0.276	57.50	4.58	23.63	0.05
W7	8.39	185.70	399.00	9.56	12.40	611.00	0.058	580.00	5.50	24.44	0.54
W8	8.36	183.60	417.00	8.67	15.20	696.00	0.073	429.00	5.49	12.18	0.17
W9	8.40	177.50	398.00	9.46	14.00	640.00	0.063	540.00	5.16	20.84	0.15
W10	8.44	191.40	365.00	8.98	16.20	624.00	0.005	615.00	4.89	6.18	0.15
W11	8.50	178.20	340.00	11.62	15.10	561.00	0.632	17.30	4.50	11.10	0.07
W12	8.66	157.50	188.10	11.29	18.30	338.00	0.672	12.10	3.73	16.01	0.01
W13	8.22	167.20	262.00	9.96	16.60	449.00	0.389	46.10	2.33	12.93	0.04
W14	8.54	150.80	258.00	12.27	19.30	478.00	0.306	22.50	9.30	11.92	0.02
W15	8.16	178.90	310.00	9.68	18.30	549.00	0.352	51.70	7.35	10.91	0.03
W16	8.25	183.80	249.00	10.34	19.40	462.00	0.301	37.20	9.47	9.90	0.02
W17	8.31	160.00	650.00	9.24	15.30	1065.00	0.644	24.00	5.59	8.78	0.09
W18	8.24	166.90	447.00	10.16	14.10	716.00	0.356	32.50	6.82	7.75	0.11
W19	8.23	136.10	467.00	10.09	13.60	740.00	0.612	44.10	7.26	7.32	0.10
W20	8.29	140.70	486.00	11.21	13.10	756.00	0.278	38.50	6.84	11.89	0.11

Table S9. Spearman rank correlations among PFAS concentrations in Weihe River.

	PFBA	PFPeA	PFHxA	PFHpA	PFOA	PFDA	PFDoDA	PFNA	PFBS	PFPeS	PFHxS	PFHpS	PFOS
**PFBA**	1.00												
**PFPeA**	**0.49***	1.00											
**PFHxA**	**0.70*****	**0.73*****	1.00										
**PFHpA**	**0.73*****	**0.56****	**0.78*****	1.00									
**PFOA**	**0.49***	**0.46***	**0.57****	**0.88*****	1.00								
**PFDA**	**0.49***	**0.45***	**0.64****	**0.54***	0.43	1.00							
**PFDoDA**	0.14	0.10	0.18	0.17	0.05	0.19	1.00						
**PFNA**	**0.71*****	0.43	**0.60****	**0.78*****	**0.76*****	0.43	0.24	1.00					
**PFBS**	**0.86*****	**0.61****	**0.79*****	**0.62****	0.32	**0.63****	0.15	**0.55***	1.00				
**PFPeS**	-0.26	-0.20	-0.06	-0.30	-0.38	-0.14	-0.14	-0.26	-0.10	1.00			
**PFHxS**	**0.84*****	**0.52***	**0.64****	**0.66****	**0.49***	**0.65****	-0.13	**0.54***	**0.78*****	-0.14	1.00		
**PFHpS**	0.38	0.33	0.34	0.38	0.34	0.38	0.08	0.38	0.22	-0.05	0.34	1.00	
**PFOS**	**0.72*****	0.43	**0.54***	**0.53***	0.35	**0.47***	0.11	**0.54***	**0.78*****	-0.14	**0.62****	0.38	1.00

*** Correlation is significant at the 0.001 level, ** Correlation is significant at the 0.01 level, and * Correlation is significant at the 0.05 level.

Supplementary figures

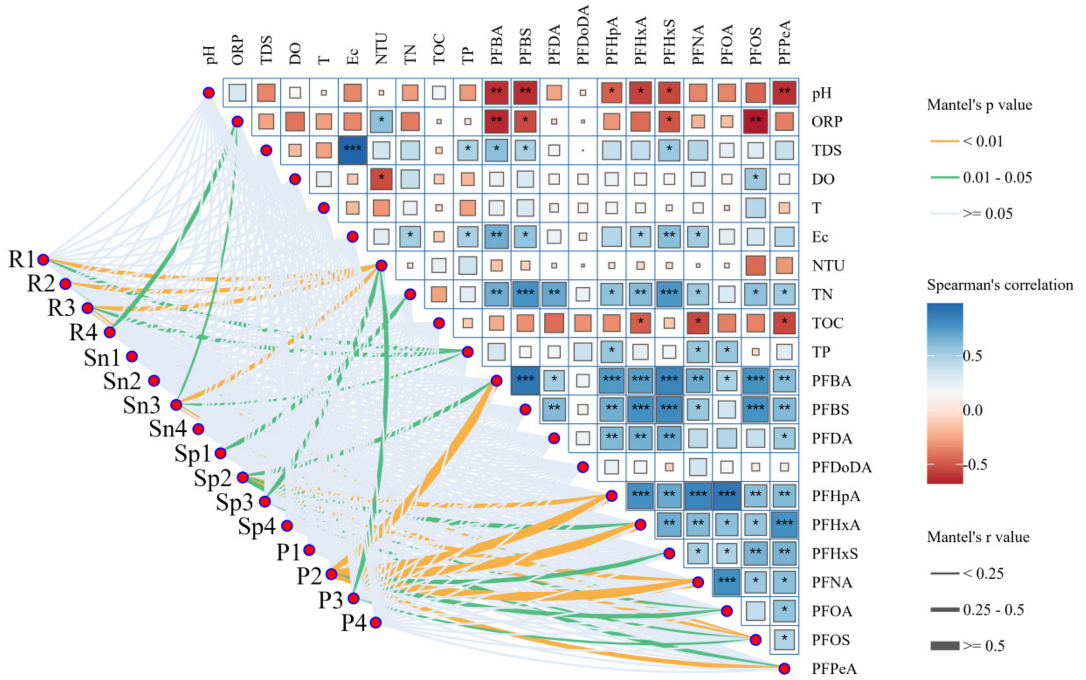

Fig. S1. Relationships between biodiversity at the taxa level and environmental variables. The network diagram illustrates the results of Mantel tests between different α-diversity and environmental variables. The width and colour of the connecting lines represent Mantel's r value and the significance level (*p*-value), respectively. The heatmap shows pairwise correlations among environmental variables. For each pair, the size and colour of a block reflect Spearman's r value, where blue denotes a positive correlation and red a negative correlation
(Note: In the figure, label characters: R = Richness, Sn = Shannon's index, Sp = Simpson's index, P = Pielou's index. Numbers 1, 2, 3, 4 represent Ciliophora, Cercozoa, Choanoflagellata, and Rotifera, respectively; *$p \leq 0.05$, **$p \leq 0.01$, ***$p \leq 0.001$).

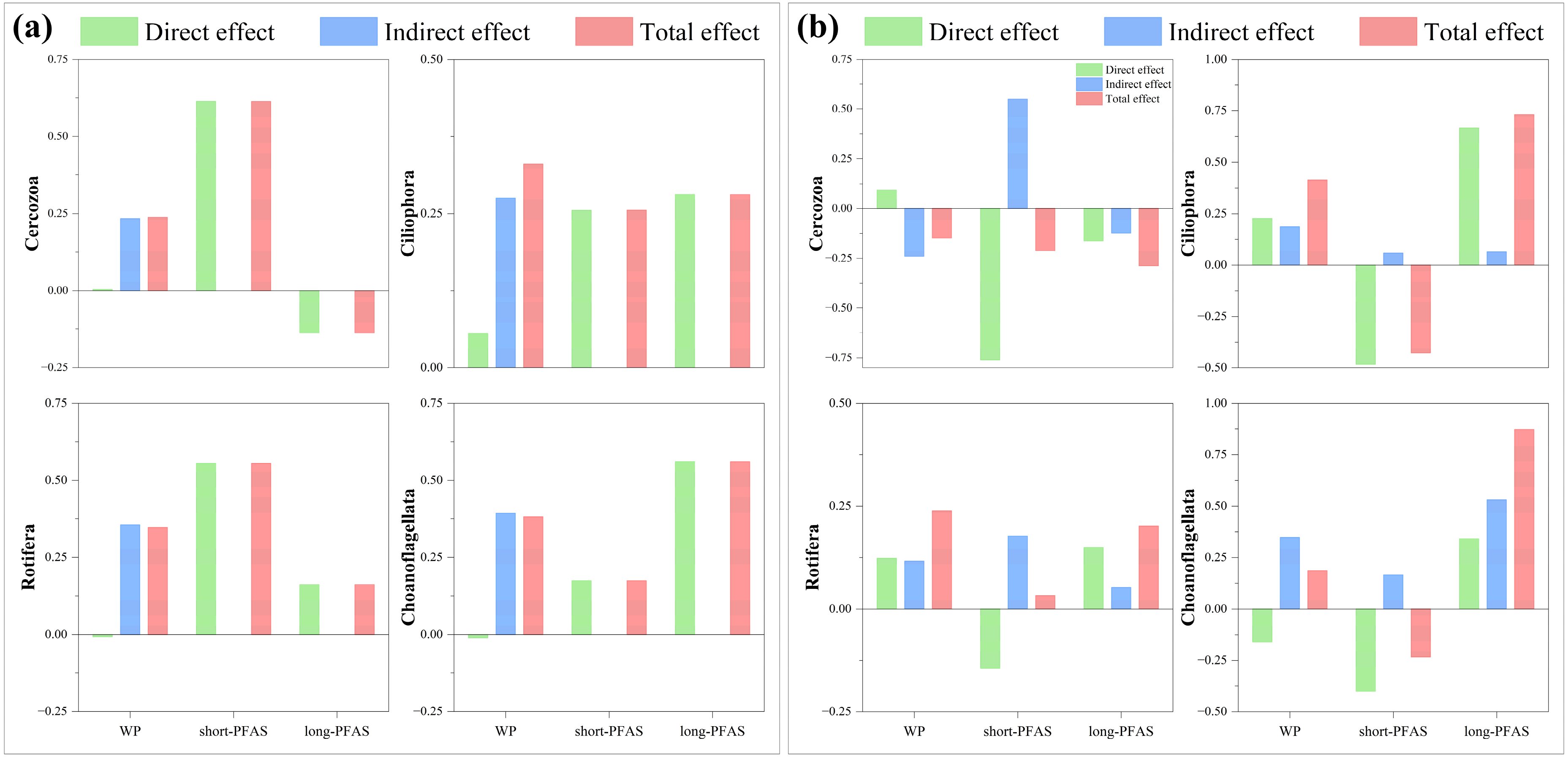

Fig. S2. Direct and indirect effects of water quality parameters (WP), short- and long-chain PFAS on zooplankton community abundance (a) and diversity(b), as estimated by partial least squares path modeling (PLS-PM).
